# Morphological, Biochemical, and Molecular Diversity Assessment of Egyptian Bottle Gourd Cultivars

**DOI:** 10.1155/2024/4182158

**Published:** 2024-01-03

**Authors:** Ehab A. Ibrahim, Haifa A. S. Alhaithloul, Sahar A. M. Shamseldin, Sara B. H. Awaly, Abd El-Latif Hesham, Mohamed F. M. Abdelkader, Mesfer M. Alqahtani, Fahad Mohammed Alzuaibr, Abdulrahman Alasmari, Noha A. Sukar, Mohamed Z. Diyasty, Mohamed A. Abdein

**Affiliations:** ^1^Department of Cross-Pollinated Vegetable Research, Horticulture Research Institute, Agricultural Research Centre, Giza 12619, Egypt; ^2^Biology Department, College of Science, Jouf University, Sakaka 2014, Aljouf, Saudi Arabia; ^3^Botany Department, Women's College for Arts, Science and Education, Ain Shams University, Cairo 11566, Egypt; ^4^Genetics Department, Faculty of Agriculture, Cairo University, Giza, Egypt; ^5^Genetics Department, Faculty of Agriculture, Beni-Suef University, Beni Suef 62521, Egypt; ^6^Department of Plant Production, College of Food and Agriculture, King Saud University, Riyadh 12372, Saudi Arabia; ^7^Department of Biological Sciences, Faculty of Science and Humanities, Shaqra University, P.O. Box 1040, Ad-Dawadimi, Shaqra 11911, Saudi Arabia; ^8^Biology Department, College of Science, Tabuk University, Tabuk 71491, Saudi Arabia; ^9^Agricultural Botany (Genetics) Department, Faculty of Agriculture (Girls), Al-Azhar University, Cairo, Egypt; ^10^Genetic Department, Faculty of Agriculture, Mansoura University, Mansoura 35516, Egypt; ^11^Seeds Development Department, El-Nada Misr Scientific Research and Development Projects, Turrell, Mansoura 35511, Egypt

## Abstract

The genetic variability and relationships between ten bottle gourd cultivars were evaluated based on morphological, biochemical, and molecular parameters. The results displayed high variability among selected cultivars in terms of photosynthetic pigments, total free amino acids, total phenol content, isozymes pattern, and protein electrophoresis. Furthermore, differences in molecular markers were revealed by the SCoT technique. The peroxidase (POD) and polyphenyl oxidase (PPO) isozymes patterns did not detect significant differences in bands among cultivars. The protein patterns revealed seventeen bands ranging from 126 to 9 kDa and five polymorphic bands representing 29.41%. On the other hand, eight SCoT primers were used to evaluate the genetic variability and relationships between the ten Egyptian bottle gourd cultivars. The results of SCoT analysis detected 44 amplicons with 50% polymorphism. In addition, the results of the phylogenetic tree that is constructed based on the similarity coefficient revealed by SCoT analysis confirm the results of biochemical analysis indicating a genetic relationship between the most efficient bottle gourd cultivars (S1 and S2 cultivars). In addition, there is a genetic relationship among the less efficient bottle gourd cultivars (S4 and S5 cultivars). These results could be beneficial to distinguish among bottle gourd cultivars in the plant breeding programs.

## 1. Introduction

Bottle gourd (*Lagenaria siceraria* (Mol.) Standl.) belongs to the Cucurbitaceae family. This crop is popularly known for the nutritional, medicinal, and economic properties of seed and edible fruits [1]. The seed is an excellent source of edible proteins, oils, vital fatty acids (such as omega 3 and 6), vitamins (A, C, and E), sterols, and antioxidants [1, 2]. Fresh fruit is cooked as food but mature dry fruit is utilized to make containers for grain and food storage, musical instruments, and garnishes [3]. Moreover, they have medicinal usage due to the presence of pharmaceutically vital bioactive compounds that can be utilized as remedies for hirsutism, acne, alopecia, and hyperseborrhea [1, 2]. In Egypt, bottle gourds are planted to produce seeds that can be used as a snack or to produce salad oil [2].

The cross-pollinating nature of this crop led to variations in fruit and seed morphology and their other traits. Seed and fruit characteristics are economic features in the development of bottle gourd cultivars that are essential for numerous industrial and domestic applications [3].

Morphological, biochemical, and molecular analysis used to detect and evaluate the phylogenetic tree among cultivars because they provide excellent information with the superior power of resolution for genetic variation analysis. Thus, morphological, biochemical, and molecular characterization is necessary to elucidate the genetic relationships among the variety of cultivars of Cucurbita [4, 5].

Although there is significant variation in the vegetative traits of bottle gourd, particularly fruit characters, it is hard to distinguish among genotypes on their morphology alone [3, 6] whereas the evaluation of genetic variation based on morphological characteristics has restrictions as the majority of phenotypes are highly affected by environmental factors and also depend on the developmental stages of the plant [3].

In contrast, isozymes are beneficial biochemical markers for the valuation of genetic diversity. They are utilized in taxonomic, evolutionary, ecological, and genetic research. Moreover, isozymes are utilized for the identification of cultivars [4].

Furthermore, SDS-PAGE (sodium dodecyl sulfate-polyacrylamide gel electrophoresis) is widely used as a rapid method to assess and identify the phylogenetic relationships between cultivars [5, 7]. However, different molecular markers based on polymorphisms are independent of environmental conditions and have proven to be forceful tools in the evaluation of genetic variability and clarification of the genetic relationships among and within cultivars and also reveal higher levels of polymorphisms [3, 6]. The evaluation of genetic variation in bottle gourd using molecular markers allows more efficient usage of plant traits in improving cultivars suitable for high yield and stability [6]. The SCoT markers (start codon targeted) technique is the type of molecular marker that depends on polymorphism in the short-conserved region flanking the start codon ATG [8–10].

The use of morphological, biochemical, and molecular markers to determine genotypic differences among Egyptian bottle gourd cultivars is an effective strategy for genetic improvement program. Therefore, the current study aimed to identify the genetic variation among ten cultivars based on differences at phenotypic, biochemical, and molecular levels using SDS-PAGE, isozymes, and SCOT molecular markers.

## 2. Materials and Methods

### 2.1. Plant Materials

Ten Egyptian bottle gourd cultivars in the S2 generation were used in this study, which were obtained from the previous research work conducted by Ibrahim [1] by using a pedigree selection program on the Egyptian bottle gourd genotypes. Seed were sown at Dakahlia, Egypt, on 3^rd^ May 2021.

The experiment was conducted in a randomized complete block design (RCBD) in three replications. Each experimental unit area was composed of 2 ridges each 3.5 meters in width and 7 meters in length, and one plant per hill 50 cm away. A recommended package of practices for bottle gourd production in Egypt was followed by Prof. Dr. Ehab A. Ibrahim (first author**)**.

Two months after planting, a random sample of three plants was taken from each experimental unit, and observations were recorded on morphological parameters like stem length (cm), the number of leaves per plant, and the node number of the first female flower. At the harvesting stage, a random sample of six plants was taken from each experimental unit, and observations were recorded on the number of fruits per plant, 100-seed weight (g), and seed yield per plant (g). Seeds were collected, washed, dried, and weighed.

### 2.2. Biochemical Parameters

#### 2.2.1. Determination of Photosynthetic Pigments

Pigments of photosynthesis (carotenoids, chlorophyll a, and b) in fresh leaves were determined according to Stino et al. [11].

#### 2.2.2. Estimation of the Total Phenolic Content

The total phenolic content of the bottle gourd samples was determined using the spectrophotometric method of Folin–Ciocalteu (FC) [12] with some modifications and was expressed as gallic acid equivalents (GAE) 100 g^−1^ FW of bottle gourd samples.

#### 2.2.3. Determination of Total Free Amino Acids

The total free amino acids in the ten bottle gourd leaf samples were measured using the spectrophotometer according to the method described by Jayaraman [13].

#### 2.2.4. Protein Electrophoresis

Sodium dodecyl sulfate-polyacrylamide gel (SDS-PAGE) was used to study the protein profiles of the ten bottle gourd cultivars. Water-soluble protein fractionation was performed on 12% (W/V) vertical slab gels using BIORAD Techware 1.5 mm according to the method of Laemmli [14] as modified by Studier [15]. The molecular weight of protein bands was relatively estimated using a wide range of molecular weights related to protein markers (Fermentas comp.). After electrophoresis, the gel was stained with Coomassie brilliant blue.

#### 2.2.5. Isozyme Electrophoresis

Extraction of peroxidase (Px) and polyphenyl oxidase (PPO) isozymes in 100 mg fresh-weight leaf samples were obtained by cellular lysis using an osmotic shock/freezing-thawing method described by Fernandes and Simoni [16]. Native PAGE (native polyacrylamide gel electrophoresis) isozyme electrophoresis was performed on 12% (W/V) slab gels described by Davis [17] to identify isozyme differences between ten bottle gourd cultivars. Then, gels were stained according to Tankesley and Rick [18] for Px and PPO isozyme. The stained gels were incubated at 37°C in dark conditions for complete staining after adding the appropriate substrates and staining solutions.

### 2.3. Molecular Analysis

#### 2.3.1. DNA Isolation

Genomic DNA was isolated from the ten bottle gourd cultivars with the QIAamp® DNA plant Mini kit according to the manufacturer's instructions. The quality of DNA was assessed by the ratio of A260/A280 absorption with a UV spectrophotometer where DNA is pure at the ratio of A260/A280 from 1.8 to 2.

#### 2.3.2. SCoT-PCR Analysis

Eight SCoT primers are used in the PCR reaction shown in Table 1. The SCoT-PCR reactions were carried out in 20 *μ*l containing 8 *μ*L d.H_2_O, 1 *μ*L genomic DNA (50 ng), 1 *μ*L primer (10 pmol), and 10 *μ*L Master Mix (GeneDireX). Amplifications were performed in a thermocycler (Biometra, Germany) using a preliminary cycle of 300 s at 94°C, 40 cycles of 60 s at 94°C, 50°C, and 60 s at 72°C, and a final cycle of 300 s at 72°C. The amplification products of SCoT-PCR were separated on 1.3% agarose gels with ethidium bromide to stain the amplicons.

### 2.4. Statistical Analysis

The morphological data obtained were statistically analyzed according to Snedecor and Cochran [19]. Differences among means were compared using the least significant difference (LSD 0.05) value 5% level.

The protein, isozyme, and SCoT banding pattern was photographed using the Gel Documentation 2000 Bio-Rad system and analyzed by GelAnalyzer3 software which scored clear amplicons as present (1) or absent (0) for each primer and entered in the form of a binary data matrix. Subsequently, the collected data were analyzed, and ten bottle gourd cultivars' coefficient of similarity was determined using Jaccard's coefficient of similarity [20] and built as a dendrogram [21] by the UPGMA method by the arithmetic average utilizing the MVSP (Multivariate Statistical Program) version 3.22 computer program.

Moreover, the potential of SCoT markers for the evaluation of genetic variation was evaluated by measuring the parameters of polymorphism information content (PIC), marker index (MI), effective multiplex ratio (EMR), and resolving power (Rp) for each primer according to [22].

## 3. Results

### 3.1. Morphological Parameters of the Bottle Gourd Cultivars

**Figure 1:**
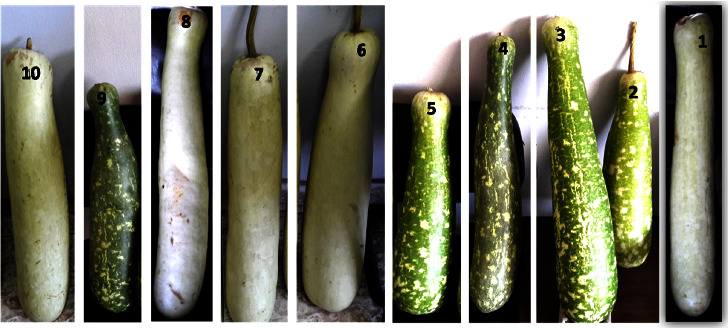
Fruit morphology of bottle gourd genotypes.

**Figure 2:**
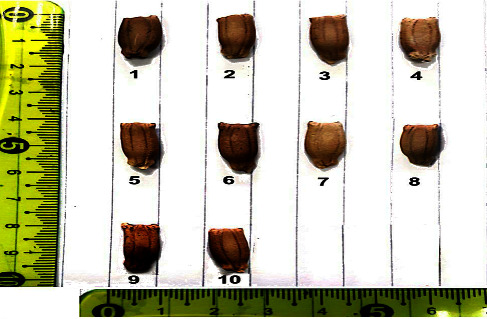
Seed morphology of bottle gourd genotypes.

The morphological data are presented in Table 2. The morphological variation in fruit shape, size, and color of the ten bottle gourd cultivars studied is presented in Figure 1, and variation in seed morphology is depicted in Figure 2.

**Figure 3:**
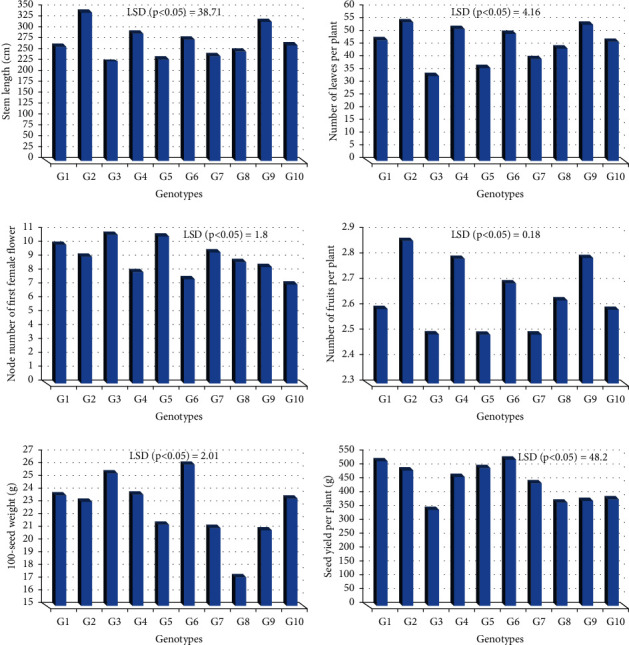
Morphological and yield traits of bottle gourd genotypes.

Figure 3 shows the means of the ten bottle gourd cultivars for each trait that was examined. The obtained data show that all cultivars showed highly significant variations for all examined variables. The mean performance gave an indicated the agricultural superiority of some lines over others. Results show that S2 and S9 possessed the highest values for stem length S1, S3, S7, and S8 possessed the lowest values. With regard to the number of leaves per plant, S2, S4, and S9 had maximum values, whereas S3 and S5 had the lowest values. Data revealed that S4, S6, S8, S9, and S10 had the lowest means of node number of first female flowers compared with other families. For the number of fruits per plant, S2, S4, S6, and S9 showed profuse fruiting compared to other lines. S3 and S6 showed a maximum 100-seed weight, while S8 showed the lowest mean. For yield per plant, S1, S2, S5, and S6 exhibited the highest values, but S3, S8, S9, and S10 exhibited the lowest values compared to other cultivars.

### 3.2. Biochemical Parameters

#### 3.2.1. Determination of Photosynthetic Pigments

Table 3 shows the total photosynthetic pigments content, as well as chlorophyll a, b, and carotenoids (*p* < 0.05) of fresh leaves of ten bottle gourd cultivars. The significant differences in photosynthetic pigments content were recorded among the ten bottle gourd cultivars. The S2 cultivar has the greatest significant content of total photosynthetic pigments (1.02 mg/100 gm) followed by S1 (1.01 mg/100 gm) while the lowest content was noticed in the S5 (0.19 mg/100 gm) whereas the S2 cultivar recorded the highest significant content of chlorophyll b and carotenoids content (0.26 mg/100 gm and 0.46 mg/100 gm, respectively) and high content of chlorophyll a (0.30 mg/100 gm) followed by the S1 cultivars recorded the highest significant content of chlorophyll a content (0.39 mg/100 gm) and the high content of chlorophyll b and carotenoids (0.24 mg/100 gm and 0.38 mg/100 gm, respectively). The S5 cultivars recorded the lowest significant content of chlorophyll b and carotenoid content (0.03 mg/100 gm and 0.06 mg/100 gm, respectively) and low content of chlorophyll a (0.10 mg/100 gm).

#### 3.2.2. Determination of Total Phenolic Content

The total phenol content (Table 3) expressed as mg GAE/100 g FW showed significant variation (*p* < 0.05). The total phenol concentration was the highest significant (*p* < 0.05) in the S1 cultivar (0.39 mg GAE/100 g) compared to the other cultivars, followed by the S8 cultivars (0.36 mg GAE/100 g), while the S5 cultivar recorded the lowest significant (*p* < 0.05) concentration of total phenol (0.11 mg GAE/100 g).

#### 3.2.3. Estimation of Total Free Amino Acids

Estimation of total free amino acids in leaves of ten Egyptian bottle gourd cultivars are presented in Table 3. A significantly higher level of total amino acid content (0.49 mg/g) was observed in the S3 cultivar than in other cultivars followed by the S2 cultivar (0.42 mg/g) and then the S1 cultivar (0.40 mg/g) whereas a significantly lower level of total amino acid content (0.01 mg/g) was observed in the S4 cultivar followed by the S5 and the S9 cultivars (0.05 mg/g).

#### 3.2.4. Protein Electrophoresis

**Figure 4:**
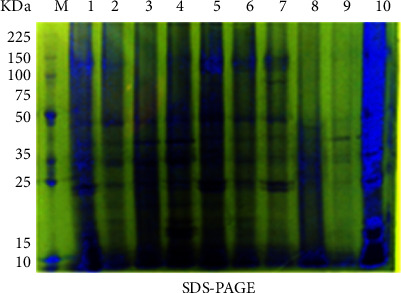
Ten Egyptian bottle gourd cultivars' protein patterns were examined using SDS-PAGE. 1: S1, 2: S2, 3: S3, 4: S4, 5: S5, 6: S6, 7: S7, 8: S8, 9: S9, 10: S10, and M: protein marker (kDa).

The SDS-PAGE proteins were used as a biochemical marker for the ten bottle gourd cultivars in this investigation. The electrophoretic protein banding pattern of the studied ten bottle gourd cultivars is demonstrated in Figure 4. SDS-PAGE scans, the recorded bands, their molecular mass in kilo Daltons, and the occurrence of bands as presence (1) or absence (0), polymorphism band, percentage of polymorphism, and biochemical markers are shown in Table 4. The bands' pattern reveals differences in the bands' molecular weight and optical density between the cultivars of bottle gourds under study.

Seventeen bands were revealed on the protein pattern of the studied ten bottle gourd cultivars with a polymorphism percentage of 29.41% (Table 4). The molecular weight of the polypeptides revealed ranged from 9 to 126 kDa. The S1, S3, S4, S5, S7, and S10 cultivars recorded the maximum number of bands (17 bands), whereas the S2 cultivar recorded the minimum number of bands (15 bands). The resulting profile comprises twelve monomorphic bands and five polymorphic bands. Among the five polymorphic bands appeared four bands in all studied cultivars except one, these bands are resolved as M^−^ and could be applied as negative biochemical markers for cultivars. These negative unique bands were detected in the cultivar S8 at 126 and 100 kDa, S6 at 23 kDa, and S2 at 11 kDa, whereas the band at MW 12 kDa was absent in the S2 and S9 cultivars.

**Figure 5:**
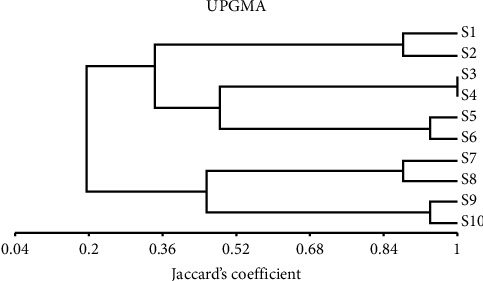
Protein pattern unweighted pair-group arithmetic (UPGMA) and Jaccard's coefficient-based similarity matrices were used to create the dendrogram of the ten bottle gourd varieties.

The relationships among the tested cultivars were described using reported results from protein profiles. The matrix of similarities between these cultivars was examined in each of the pair-wise groups. The relationships of similarity based on protein analysis ranged from 1 to 0.765 (Table 5). The lowest similarity index (0.765) was scored among S2 and S8 cultivars. Two main categories were offered by the dendrogram, with the first category split into two subcategories, the first subcategory containing S1 and S2 cultivars. While the second subcategory was split into two groups, the first group contained S3 and S4 cultivars but the second group included S5 and S6 cultivars. Also, the second category was split into two subcategories; the first subcategory consists of S7 and S8 cultivars, whereas the second subcategory includes S9 and S10 cultivars (Figure 5).

#### 3.2.5. Analysis of Isozymes Banding Patterns

**Figure 6:**
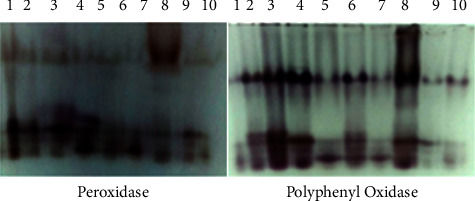
Isozyme analysis for ten Egyptian bottle gourd cultivars. 1: S1, 2: S2, 3: S3, 4: S4, 5: S5, 6: S6, 7: S7, 8: S8, 9: S9, and 10: S10.

Ten cultivars of Egyptian bottle gourd were tested for two isozymes, including peroxidase (POD) and polyphenyl oxidase (PPO) enzymes to investigate genetic diversity (Figure 6). Two isozymes appeared in five bands (*R*_*m*_ of 0.3, 0.6, 0.65, 0.7, and 0.75) of which one (*R*_*m*_ of 0.6) was polymorphic representing 20%. This band was absent for all cultivars except S1 and S2 cultivars. The remaining bands were all monomorphic. In each enzyme system, three isozyme banding patterns were identified (Tables 6 and 7).

**Figure 7:**
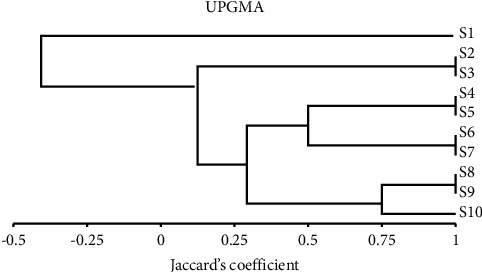
Isozyme pattern unweighted pair-group arithmetic (UPGMA) and Jaccard's coefficient-based similarity matrices were used to create the dendrogram of the ten bottle gourd varieties.

Jaccard similarity coefficient was applied to construct the matrix of similarity among ten Egyptian bottle gourd cultivars based on isozyme types. The matrix of similarities between these cultivars was tested in each of the pair-wise groups. The relationships of similarity based on isozymes ranged from 1 to 0 (Table 8). The cultivar S1 showed a significantly lower similarity (0) with all the other cultivars. The dendrogram provided two main categories; the first category contains the S1 cultivar. Whereas the second category was split into two subcategories, the first subcategory consists of S2 and S3 cultivars. The second subcategory was split into two groups, the first group was split into two subgroups, the first subgroup contained S4 and S5 cultivars but the second subgroup included S6 and S7 cultivars. While the second group was split into two subgroups, the first subgroup contained S8 and S9 cultivars but the second subgroup included S10 cultivars (Figure 7).

The correlations among the tested cultivars were described using the reported results from the combined protein-isozyme data. The relationships of similarity ranged from 1 to 0.778 (Table 9). The highest value of similarities was 1 among the S3, S4, S5, S7, and S10 cultivars and of them while the lowest similarity value was 0.778 between S2 and S8 cultivars.

**Figure 8:**
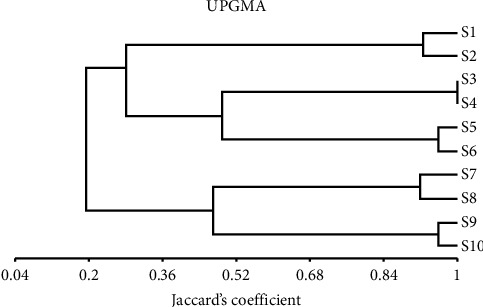
Using pooled isozyme-protein data unweighted pair-group arithmetic (UPGMA) and similarity matrices calculated by Jaccard's coefficient; the dendrogram of the ten bottle gourd cultivars was generated.

Using arithmetic averages, dendrograms were created using the unweighted pair-group approach (UPGMA). The dendrogram sectioned all the cultivars based on the protein-isozyme combined data (Figure 8). The pooled dendrogram was similar to the protein dendrogram. The dendrogram provided two main categories; the first category was split into two subcategories, the first subcategory containing S1 and S2 cultivars. While the second subcategory was split into two groups, the first group contained S3 and S4 cultivars but the second group included S5 and S6 cultivars. Also, the second category was split into two subcategories, the first subcategory consists of S7 and S8 cultivars, whereas the second subcategory includes S9 and S10 cultivars.

### 3.3. Molecular Analysis

**Figure 9:**
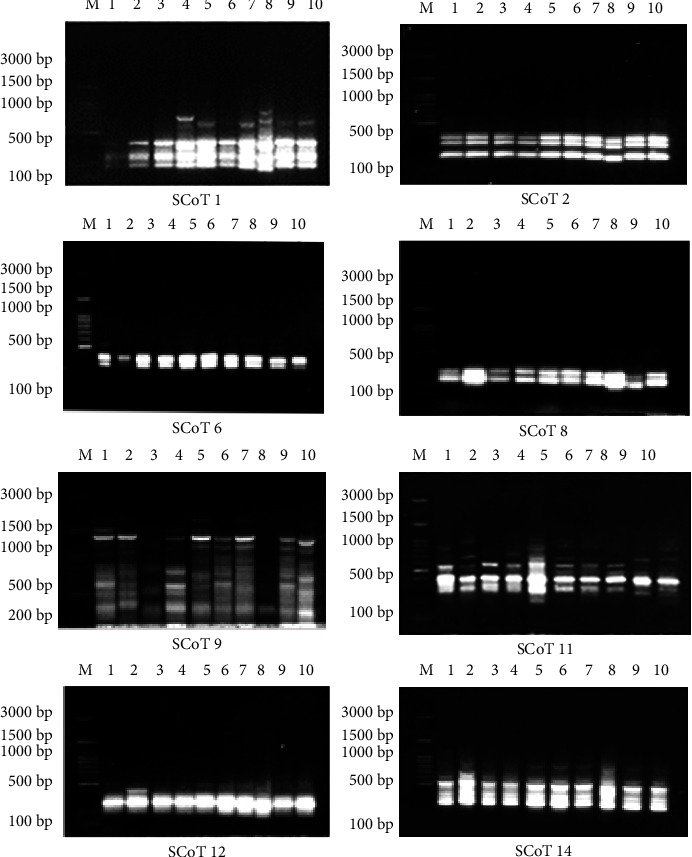
Banding patterns of SCoT-PCR products for ten examined bottle gourd cultivars produced with eight primers. M: 100 bp ladder DNA marker, 1: S1, 2: S2, 3: S3, 4: S4, 5: S5, 6: S6, 7: S7, 8: S8, 9: S9, and 10: S10.

The eight SCoT primers applied in this study showed the amplification results which is shown in in Table 10. The amplification profiles were inspected for the presence of polymorphisms between the ten tested bottle gourd cultivars, and the eight primers generated high repeatable and scorable bands (Figure 9). The ten studied bottle gourd cultivars yielded 44 amplified bands with a range of 210 to 1680 bp. The amplification bands generated from the tested primers ranged from 11 bands for SCoT-9 to 3 bands for SCoT-2, SCoT-6, and SCoT-8. The total number of monomorphic and polymorphic bands was 22 bands. The polymorphism percentages ranged between 0% with SCoT-2 and 66.66% with SCoT-8 with an average of 45.15% as shown in Table 10.

Furthermore, the parameters of the genetic varieties for the investigated populations and primers were determined with the informativeness of the primers. The values of polymorphism information content (PIC) for the SCoT markers ranged from 0 by SCoT-2 to 0.25 SCoT-11 with an average of 0.14. The amplification profile was detected by the three SCoT primers SCoT-11, SCoT-1, and SCoT-9 which showed higher PIC values (0.25, 0.22, and 0.21, respectively), based on computed PIC, produced extremely informative patterns, and has more potential for further study shown in Table 10. Moreover, the highest EMR value (6.05) was obtained by SCoT-9, while the lowest value (1.38) was obtained by primer SCoT-8 with an average of 3.06. The following RP values were approximately (3.17) according to SCoT-8 to (14) according to SCoT-9 by an average of 6.88. Also, MI values range from 0 according to primer SCoT-2 to 1.25 according to SCoT-9 by an average of 0.47.

Each cultivar's banding patterns were distinct according to the SCoT fingerprints.  Table 11 displayed the estimated genotype-specific markers for ten cultivars of bottle gourd. There were six genotype-specific markers in total. The S2 cultivar recorded two positive unique markers of SCoT genotype-specific markers by SCoT-9 at 460 bp and SCoT-12 at 415 bp. Moreover, one positive unique SCoT marker scored with the S8 cultivar by SCoT-1 at 835 bp and the S9 cultivar by SCoT-8 at 400 bp. Additionally, one negative unique SCoT marker scored with the S1 cultivar by SCoT-14 at 830 bp and the S3 cultivar by SCoT-12 at 500 bp. While the cultivars S4, S5, S6, S7, and S10 did not record any unique SCoT marker. These bands may be utilized to discriminate the tested cultivars.

The genetic similarity coefficient among ten bottle gourd cultivars ranged from the lowest similarity (0.675) between the S5 and S9 cultivars to the highest similarity (0.889) between the S5 and the S7, as shown in Table 12.

**Figure 10:**
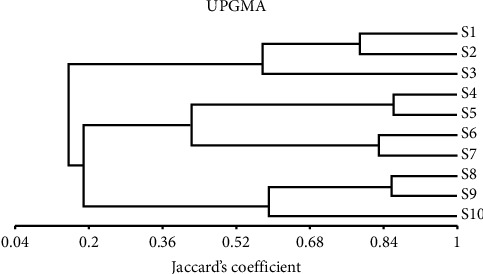
The SCoT pattern unweighted pair-group arithmetic (UPGMA) and Jaccard's coefficient-based similarity matrices were used to create the dendrogram of the ten bottle gourd varieties.

Figure 10 displays the phylogenetic tree constructed using the similarity coefficient generated from the SCoT markers. The ten bottle gourd cultivars were split into two main categories. The first main category was split into two subcategories. The first subcategory included the S1 and the S2 cultivars, whereas the second subcategory included the S3 cultivar. Furthermore, the second main category was split into two subcategories. The first subcategory was split into two groups. The first group included the S4 and the S5 cultivars, while the second group included the S6 and the S7 cultivars. Additionally, the second subcategory was split into two groups. The first group included the S8 and the S9 cultivars, whereas the second group included the S10 cultivar.

## 4. Discussion

The highly significant differences detected among the means of the ten cultivars of bottle gourd for all morphological studied traits indicated that there was a wide range of variation among the studied genotypes for all studied traits, which provides an opportunity for selecting suitable genotypes with better performance for the studied traits. This result also implied that these populations of bottle gourd genotypes would respond positively to selection. Similar results were obtained by Quamruzzaman et al. [23].

Achieving sustainable farming and optimal crop production requires monitoring the physiological status of bottle gourd cultivars over time. The photosynthetic pigments content including chlorophyll a, b, and carotenoids have been frequently used to evaluate plants' health under various environmental conditions [24]. Chlorophyll a and b are photosynthetic pigments responsible for the green color of plants. Carotenoids are yellow and orange pigments that take part in photosynthesis by protecting photosystems [24, 25]. In human nutrition, photosynthetic pigments have a rich color making products more desirable for consumption as well as various functions in human health, such as antioxidant activity, improvements in cognitive function, anticancer properties, immunomodulation activities carotenoids as pro-vitamin A activity, and prevention of degenerative diseases as well as eye and cardiovascular health [24, 25]. In this study, ten bottle gourd cultivars exhibited distinct photosynthetic pigments content, as well as chlorophyll a, b, and carotenoids (*p* < 0.05) in fresh leaves (Table 3) were used to assess the genetic diversity and plants' health. Significant differences in photosynthetic pigment content were recorded among the ten bottle gourd cultivars. The S2 cultivar recorded the highest significant content of total photosynthetic pigments (1.02 mg/100 gm) followed by S1 (1.01 mg/100 gm) while the lowest content was noticed in the S5 (0.19 mg/100 gm).

The current research provides the determined amounts of total free amino acids (the primary metabolites) and total phenol content (the secondary metabolites) present in the leaves of ten Egyptian bottle gourd cultivars. This may provide information on the nutritional quality and its biological functions [26].

Phenolic compounds are one of the main antioxidant constituents of natural products. Data presented in Table 3 indicate that total phenolic content in bottle gourd cultivars revealed a significant difference (*p* < 0.01) with a range of 0.39 mg/100 g in the S1 cultivar to 0.11 mg/100 g in the S5 cultivar. Some studies may find a higher level of total phenolics than those observed in our experiments. These differences may be due to various agronomic factors such as cultivars, growing conditions, harvest time, and weather. Some researchers also demonstrated that total phenolics content strictly depends on the fruit ripening stage, as there is a higher concentration in green and intermediate ripening stages, reducing in full fruits [27].

Amino acids are a kind of nitrogenous compound that plays a vital and varied role in metabolism and have attracted large attention in feeds, foods, and nutrients. Amino acids are fundamental units and essential protein components and are involved in furthering the biosynthesis of polyamines, porphyrins, nitric oxide, glycoproteins, and neurotransmitters [28]. Data presented in Table 3 indicate that total free amino acids content in bottle gourd cultivars showed a significant difference (*p* < 0.02) with a range of 0.49 mg/g in the S2 cultivar to 0.01 mg/g in the S4 cultivar. Nutrition research reveals that amino acids can alter gene expression and improve skeletal muscle growth and the small intestine. The content of free amino acids is very significant in the evaluation of a protein-rich diet [28].

In the current study, the results of the quantitative determination of total photosynthetic pigments included chlorophyll a, b, and carotenoids, total free amino acids as primary metabolites, and total phenolic concentration as secondary metabolites in the leaves of ten Egyptian bottle gourd cultivars, we found the most efficient bottle gourd cultivars determined on the bases of their quantitative production is the S1 cultivar (0.39 mg/100 g, 0.24 mg/100 g, 0.38 mg/100 g, 0.39 mg/100 g, and 0.40 mg/g, respectively) and the S2 cultivar (0.30 mg/100 g, 0.26 mg/100 g, 0.46 mg/100 g, 0.17 mg/100 g, and 0.42 mg/g, respectively). While the lowest efficient bottle gourd cultivars determined on the bases of their quantitative production is the S5 cultivar (0.10 mg/100 g, 0.03 mg/100 g, 0.06 mg/100 g, 0.11 mg/100 g, and 0.05 mg/g, respectively), the S4 cultivar (0.12 mg/100 g, 0.05 mg/100 g, 0.09 mg/100 g, 0.16 mg/100 g, and 0.01 mg/g, respectively), and the S9 cultivar (0.06 mg/100 g, 0.07 mg/100 g, 0.13 mg/100 g, 0.19 mg/100 g, and 0.05 mg/g, respectively).

Biochemical markers like isozymes and proteins have evolved as a result of the restrictions of morphological markers [29]. However, the genetic environment of each taxon affects how proteins attach to each other, and as a result, it is advantageous to examine the genetic diversity among cultivars [29]. SDS-PAGE is an efficient tool for studying the plant genetic variety in a relatively short period. SDS-PAGE analysis can be readily used for a variety of purposes, such as biosystematics research, germplasm differentiation, varietal validation, determination of phylogenetic interactions among different cultivars, and the production of relevant information to balance measurement. SDS-PAGE analysis has been successful in evaluating the relationship between cultivars [29].

In this study, SDS-PAGE and two isozymes, including peroxidase (POD) and polyphenyl oxidase (PPO) enzymes were used successfully to identify relationships between ten bottle cultivars. These ten cultivars had higher polymorphism than the majority of the isozymes (29.41%), according to SDS-PAGE. A higher polymorphism has also been detected in other species. For instance, nine cucumber cultivars demonstrated 95.65% polymorphism, according to Habiba et al. [29]. While isozyme pattern is an extremely effective genetic marker that costs less than DNA-based markers and may be detected by faster and simpler laboratory procedures. Some enzymes are more advantageous than others in variety and polymorphism analyses, according to Habiba et al. [29]. For instance, in this study, peroxidase (POD) and polyphenyl oxidase (PPO) enzymes produced 5 bands of which one was polymorphic (*R*_*m*_ of 0.6). These enzymes showed low suitability for diversity analysis in ten cultivars of Egyptian bottle gourd.

When describing phylogenetic relationships at the molecular level, molecular markers such as SCoT are used. SCoT is one of the molecular marker systems based on a preserved sequence of genes surrounding the start codon of translation (ATG). SCoT uses one primer constructed to anneal the ATG surrounding regions into two strands of DNA. The SCoT technique is a dominant marker such as RAPDs. It is possible that some SCoT markers may be codominant due to deletion-insertion mutations and may be used for genetic analysis and QTL mapping [10].

In this study, the genetic markers as biochemical and molecular markers showed differences in the level of genetic similarity among the ten Egyptian bottle gourd cultivars. This could be the result of the various processes of detection of polymorphism by the proteins, isozyme, and SCoT markers. Proteins and isozymes are the product of gene expression, whereas SCoT polymorphism results in differences in the nucleotide sequence of DNA in a primer attached to random sites and the difference in DNA length between different primer binding sites [30]. The valuation of SCoT markers was determined in ten Egyptian bottle gourd cultivars. The amplification results revealed that the cultivars by the SCoT mark assay detected good repeatability and high polymorphism. Moreover, SCoT markers have been efficiently and effectively applied to detect variation between cultivars as eleven SCoT primers were applied to estimate the genetic variety of six Egyptian soybeans [5] and ten SCoT primers were used to evaluate ten barley lines belonging to *Hordeum vulgare* L. [9]. Similarly, Samarina et al. [10] estimated the efficiency of 36 new SCoT markers for the distinction of genetic variance in chrysanthemums.

Table 10 shows the polymorphism percentages ranged between 66.66% by SCoT-8 and 0% by SCoT-2 with an average of 50%. This result is in harmony with the polymorphism percentages ranging between 28.57% with SCoT-22 and 66.66% with SCoT-4 [5]. Abdein [4] used six SCoT primers to evaluate the genetic variance between 16 pumpkins whereas the polymorphism percentages ranged between 93.94% with SCoT-1 and 100% with SCoT8, SCoT11, SCoT12, and SCoT14.

Eight SCoT primers were investigated for their ability to detect polymorphic patterns in ten tested bottle gourd cultivars. Furthermore, to estimate the efficiency of the markers, the parameters polymorphism information content (PIC), marker index (MI), effective multiplex ratio (EMR), and resolving power (Rp) were analyzed to measure genetic variation among the ten tested bottle gourd cultivars.

These parameters' value of the molecular marker is crucial for germplasm valuation, the building of gene banks, gene mapping, and molecular breeding. Moreover, these parameters' value is measured by the ability of the marker to create a polymorphism in the cultivars depending on the number of exposed alleles and the frequency of distribution; it is therefore equal to genetic variation [31].

In general, the SCoT-8 primer revealed the lowest TBN (3) and values for EMR (1.38) and RP (3.17), while the SCoT-9 primer revealed the highest TBN (11) and values for all parameters (PIC (0.21), EMR (6.05), IM (1.25), and RP (14)) followed by SCoT-11 and SCoT-1 (TBN (7), PIC (0.25 and 0.22, respectively)), EMR (3.43 and 3.02, respectively), MI (0.86 and 0.67, respectively), and RP (8.33 and 7.33, respectively). These primers provide a greater possibility for future study, enabling the investigation of more cultivars with fewer primers. Our findings demonstrated that SCoT markers have greater labeling efficiency and a strong polymorphism detection ability. Similarly, Zhao et al. [8] evaluated the study of the genetic variation of 33 samples of *Paris polyphylla*.

Calculating the PIC of the eight selected SCoT primers' loci and the polymorphism level among the ten bottle gourd cultivars were subjected to testing allowed for the expression of their differentiation power (DP) to measure genetic variation among these cultivars. The maximum PIC value for dominant markers is 0.5 [10]. PIC values were observed in other investigations, such as 0.38 by the RAPD marker [32] and 0.44 by the SCoT marker [5]. Therefore, PIC parameters are applied to verify their benefits in fingerprint detection [33].

Table 12 shows the highest similarity value of 0.889 was scored among the S5 and the S7, while the S5 and S9 cultivars had the lowest value (0.675). This result is consistent with other studies that used a SCoT marker to construct the phylogenetic relationships of six Egyptian soybean cultivars, with the highest similarity value of 0.83 being scored between the Giza82 and Giza35 cultivars and the lowest value being 0.646 between the Giza82 and Giza21 cultivars [5]. The phylogenetic relationships between ten *Hordeium vulgare* genotypes were built by Aboulila and Mansour [34] using the SCoT marker, and they concluded that the SCoT marker is a useful tool for creating new *Hordeium vulgare* fingerprints. Moreover, Mohamed et al. [35] application of a SCoT marker allowed them to identify and distinguish between 14 different wheat cultivars, and they then divided these cultivars into numerous clusters by the dendrogram that resulted. Abdein et al. [36] used the SCoT marker on 14 summer squash genotypes. Also, Osman and Abdein [37] used molecular studies between six species of *Plantago*.

Based on the similarity coefficient obtained from the tested SCoT markers, the phylogenetic tree is constructed (Figure 10). These results confirmed the results of biochemical parameters indicating a genetic relationship between the most efficient bottle gourd cultivars (the S1 and the S2 cultivars). In addition, there is a genetic relationship among the less efficient bottle gourd cultivars (the S4 and the S5 cultivars).

## 5. Conclusion

This study revealed that high variance for morphological, biochemical, and molecular profiles according to different regions. The genetic resources description is a vital key for the management of plant breeding programs and gene banks. The ten Egyptian bottle gourd cultivars estimated in this study showed high diversity for some fruit parameters such as fruit shape, size, and color (Figure 1), seed morphology (Figure 2) and yield traits such as the number of leaves per plant, number of fruits per plant, stem length, seeds yield per plant, the 100-seed weight, and node number of the first female flower (Figure 3). Moreover, there is high diversity among all cultivars in the amounts of photosynthetic pigments including chlorophyll a, b, and carotenoids, with total free amino acids as primary metabolites, and total phenol content as secondary metabolites (Table 3). Furthermore, the results of protein (Figure 4 and Table 4), isozymes (Tables 6 and 7, and Figure 6), and SCoT analysis showed differences in the degree of genetic variation among all cultivars (Table 10 and Figure 9). The high genetic variety found could be used in plant breeding programs to identify new cultivars and to provide related information for biodiversity conservation. The results of the phylogenetic tree that are constructed based on the similarity coefficient revealed by SCoT analysis confirm the results of biochemical parameters (Figure 5, Table 5, Figure 7, Table 8, Figure 9, and Table 9) indicating a genetic relationship between the most efficient bottle gourd cultivars (the S1 and the S2 cultivars) (Table 11). In addition, there is a genetic relationship among the less efficient bottle gourd cultivars (the S4 and the S5 cultivars) (Table 12 and Figure 10). These results could be beneficial to distinguish among bottle gourd cultivars in the plant breeding programs.

## Figures and Tables

**Table 1:** Sequences of SCoT primers that were used to compare the genetic variance between the ten bottle gourd cultivars.

Name	Primer sequence
SCoT 1	5′ACGACATGGCGACCACGC3′
SCoT 2	5′ACCATGGCTACCACCGGC3′
SCoT 6	5′CAATGGCTACCACTACAG3′
SCoT 8	5′ACAATGGCTACCACTGAG3′
SCoT 9	5′ACAATGGCTACCACTGCC3′
SCoT 11	5′ACAATGGCTACCACTACC3′
SCoT 12	5′CAACAATGGCTACCACCG3′
SCoT 14	5′ACCATGGCTACCAGCGCG3′

**Table 2:** Morphological description of fruits of bottle gourd genotypes.

Genotypes	L fruit (cm)	D fruit (cm)	Fruit color	Speckles
S1	65.1	11.0	Light green	Absent
S2	40.7	11.3	Dark	Present
S3	59.2	11.0	Dark	Present
S4	55.8	11.3	Very dark	Present
S5	44.0	12.4	Dark	Present
S6	61.1	13.0	Very light	Absent
S7	60.3	10.3	Light green	Absent
S8	60.2	8.3	Light green	Absent
S9	46.4	10.5	Very dark	Present
S10	58.2	11.6	Light green	Absent

L: length and D: diameter.

**Table 3:** Total chlorophyll (a, b), *β*-carotene, total photosynthetic pigments, total phenols contents, and total amino acids of leaves for ten bottle gourd cultivars.

Cultivars	Chlorophyll a mg/100 gm fresh weight	Chlorophyll b mg/100 gm fresh weight	Carotenoid mg/100 gm fresh weight	Total pigments mg/100 gm fresh weight	Total phenols mg GAE/100 g	Total amino acids mg/g
S1	0.39	0.24	0.38	1.01	0.39	0.40
S2	0.30	0.26	0.46	1.02	0.17	0.42
S3	0.16	0.07	0.14	0.37	0.24	0.49
S4	0.12	0.05	0.09	0.26	0.16	0.01
S5	0.10	0.03	0.06	0.19	0.11	0.05
S6	0.19	0.11	0.15	0.45	0.14	0.08
S7	0.20	0.12	0.18	0.50	0.21	0.16
S8	0.11	0.19	0.13	0.43	0.36	0.10
S9	0.06	0.07	0.13	0.26	0.19	0.05
S10	0.13	0.24	0.29	0.66	0.19	0.39
LSD 0.05	0.05	0.05	0.05	0.05	0.01	0.02

**Table 4:** Ten Egyptian bottle gourd cultivars' protein patterns were examined using SDS-PAGE, their molecular mass in kDa, the occurrence of bands as presence (1) or absence (0), polymorphism band, percentage of polymorphism, and biochemical markers.

Band no.	M.W. (kDa)	Cultivars										Polymorphism	BM^a^
		S1	S2	S3	S4	S5	S6	S7	S8	S9	S10		
1	126	1	1	1	1	1	1	1	0	1	1	Polymorphic	M^−^
2	115	1	1	1	1	1	1	1	1	1	1	Monomorphic	
3	100	1	1	1	1	1	1	1	0	1	1	Polymorphic	M^−^
4	61	1	1	1	1	1	1	1	1	1	1	Monomorphic	
5	46	1	1	1	1	1	1	1	1	1	1	Monomorphic	
6	40	1	1	1	1	1	1	1	1	1	1	Monomorphic	
7	35	1	1	1	1	1	1	1	1	1	1	Monomorphic	
8	33	1	1	1	1	1	1	1	1	1	1	Monomorphic	
9	26	1	1	1	1	1	1	1	1	1	1	Monomorphic	
10	23	1	1	1	1	1	0	1	1	1	1	Polymorphic	M^−^
11	20	1	1	1	1	1	1	1	1	1	1	Monomorphic	
12	17	1	1	1	1	1	1	1	1	1	1	Monomorphic	
13	15	1	1	1	1	1	1	1	1	1	1	Monomorphic	
14	12	1	0	1	1	1	1	1	1	0	1	Polymorphic	
15	11	1	0	1	1	1	1	1	1	1	1	Polymorphic	M^−^
16	10	1	1	1	1	1	1	1	1	1	1	Monomorphic	
17	9	1	1	1	1	1	1	1	1	1	1	Monomorphic	
Total		17	15	17	17	17	16	17	15	16	17	% of polymorphism = 29.41%	

BM^a^, biochemical markers including either the presence (M^+^) or absence (M^−^) of a given band in the genotype.

**Table 5:** The similarity matrix between ten bottle gourd cultivars as calculated by Jaccardʼs coefficient was detected with protein markers.

	S1	S2	S3	S4	S5	S6	S7	S8	S9	S10
S1	1.000									
S2	0.882	1.000								
S3	1.000	0.882	1.000							
S4	1.000	0.882	1.000	1.000						
S5	1.000	0.882	1.000	1.000	1.000					
S6	0.941	0.824	0.941	0.941	0.941	1.000				
S7	1.000	0.882	1.000	1.000	1.000	0.941	1.000			
S8	0.882	0.765	0.882	0.882	0.882	0.824	0.882	1.000		
S9	0.941	0.938	0.941	0.941	0.941	0.882	0.941	0.824	1.000	
S10	1.000	0.882	1.000	1.000	1.000	0.941	1.000	0.882	0.941	1.000

**Table 6:** Isozyme banding patterns of ten Egyptian bottle gourd cultivars using peroxidase enzyme.

Peroxidase groups	Relative mobility	S1	S2	S3	S4	S5	S6	S7	S8	S9	S10
POD 1	0.3	1^++^	1^++^	1^++^	1^++^	1^++^	1^+^	1^+^	1^+++^	1^++^	1^++^
POD 2	0.6	1^+++^	1^++^	0	0	0	0	0	0	0	0
POD 3	0.65	1^+++^	1^++^	1^+++^	1^+++^	1^++^	1^++^	1^++^	1^++^	1^+++^	1^++^
POD 4	0.7	1^++^	1^++^	1^++^	1^+++^	1^++^	1^+^	1^+^	1^+^	1^++^	1^+^
POD 5	0.75	1^+++^	1^++^	1^++^	1^+++^	1^++^	1^+^	1^+++^	1^+^	1^++^	1^++^

^+^low density, ^++^medium density, ^+++^high density of band.

**Table 7:** Isozyme banding patterns of ten Egyptian bottle gourd cultivars using polyphenyl oxidase enzymes.

Polyphenyl oxidase groups	Relative mobility	S1	S2	S3	S4	S5	S6	S7	S8	S9	S10
PPO 1	0.3	1^++^	1^+++^	1^+++^	1^++^	1^+++^	1^++^	1^++^	1^+++^	1^++^	1^++^
PPO 2	0.6	1^++^	1^+++^	0	0	0	0	0	0	0	0
PPO 3	0.65	1^+^	1^++^	1^+++^	1^+++^	1^++^	1^++^	1^+^	1^+++^	1^+++^	1^+^
PPO 4	0.7	1^+^	1^++^	1^+++^	1^++^	1^+^	1^++^	1^++^	1^++^	1^+^	1^+^
PPO 5	0.75	1^+++^	1^+++^	1^+++^	1^+++^	1^+++^	1^+++^	1^+++^	1^+++^	1^+^	1^+^

^+^low density, ^++^medium density, ^+++^high density of band.

**Table 8:** The similarity matrix among studied ten Egyptian bottle gourd cultivars as calculated by Jaccardʼs coefficient was detected with isozyme markers.

	S1	S2	S3	S4	S5	S6	S7	S8	S9	S10
S1	1.000									
S2	0.000	1.000								
S3	0.000	1.000	1.000							
S4	0.000	0.800	0.800	1.000						
S5	0.000	0.800	0.800	1.000	1.000					
S6	0.000	0.800	0.800	1.000	1.000	1.000				
S7	0.000	0.800	0.800	1.000	1.000	1.000	1.000			
S8	0.000	0.800	0.800	1.000	1.000	1.000	1.000	1.000		
S9	0.000	0.800	0.800	1.000	1.000	1.000	1.000	1.000	1.000	
S10	0.000	0.800	0.800	1.000	1.000	1.000	1.000	1.000	1.000	1.000

**Table 9:** Jaccard's similarity coefficient indexes between ten Egyptian bottle gourd cultivars based on pooled isozyme-protein.

	S1	S2	S3	S4	S5	S6	S7	S8	S9	S10
S1	1.000									
S2	0.926	1.000								
S3	0.926	0.852	1.000							
S4	0.926	0.852	1.000	1.000						
S5	0.926	0.852	1.000	1.000	1.000					
S6	0.889	0.815	0.960	0.960	0.960	1.000				
S7	0.926	0.825	1.000	1.000	1.000	0.960	1.000			
S8	0.852	0.778	0.920	0.920	0.920	0.880	0.920	1.000		
S9	0.889	0.885	0.960	0.960	0.960	0.920	0.960	0.880	1.000	
S10	0.926	0.852	1.000	1.000	1.000	0.960	1.000	0.920	0.960	1.000

**Table 10:** PCR amplicons obtained from SCoT markers of ten bottle gourd cultivars, total band number (TBN), number of the monomorphic band (MBN), number of the polymorphic band (PBN), percentage of polymorphism (P %), unique band (UB), polymorphism information content (PIC), effective multiplex ratio (EMR), marker index (MI), resolving power (RP), and band size range (BSR).

No.	Primer name	TBN	MBN	PBN	P %	UB	PIC	EMR	MI	RP	BSR (bp)
1	SCoT-1	7	3	4	57.14	1	0.22	3.02	0.67	7.33	835-175
2	SCoT-2	3	3	0	0	0	0	2.5	0	5	390-200
3	SCoT-6	3	2	1	33.33	0	0.11	1.7	0.18	3.67	530-300
4	SCoT-8	3	1	2	66.66	1	0.17	1.38	0.23	3.17	400-215
5	SCoT-9	11	4	7	63.63	1	0.21	6.05	1.25	14	1680-300
6	SCoT-11	7	3	4	57.14	0	0.25	3.43	0.86	8.33	1325-215
7	SCoT-12	4	2	2	50	1	0.09	2.35	0.21	5	500-210
8	SCoT-14	6	4	2	33.33	1	0.08	4.04	0.34	8.5	830-280
	Total	44	22	22		5					
	Average				45.15		0.14	3.06	0.47	6.88	

**Table 11:** Bottle gourd cultivars and their specific SCoT markers.

Genotypes	Markers				Total marker/variety
	Unique positive markers		Unique negative markers		
	Primer	Band size (bp)	Primer	Band size (bp)	
S1			SCoT 14	(830)	1
S2	SCoT 9	(460)			2
	SCoT 12	(415)			
S3			SCoT 12	(500)	1
S8	SCoT 1	(835)			1
S9	SCoT 8	(400)			1
Total					6

**Table 12:** The Jaccard coefficient-based similarity matrix between ten bottle gourd cultivars as detected by SCoT markers.

	S1	S2	S3	S4	S5	S6	S7	S8	S9	S10
S1	1.000									
S2	0.789	1.000								
S3	0.771	0.778	1.000							
S4	0.737	0.700	0.722	1.000						
S5	0.763	0.725	0.703	0.861	1.000					
S6	0.800	0.757	0.788	0.853	0.829	1.000				
S7	0.811	0.816	0.703	0.811	0.889	0.829	1.000			
S8	0.757	0.763	0.743	0.757	0.737	0.771	0.833	1.000		
S9	0.784	0.744	0.771	0.737	0.675	0.703	0.763	0.857	1.000	
S10	0.842	0.800	0.737	0.842	0.868	0.811	0.868	0.816	0.795	1.000

## Data Availability

Relevant data applicable to this research are within the paper.
